# An efficient protocol for the purification and labeling of entire yeast septin rods from *E.coli* for quantitative *in vitro* experimentation

**DOI:** 10.1186/1472-6750-13-60

**Published:** 2013-07-26

**Authors:** Christian Renz, Nils Johnsson, Thomas Gronemeyer

**Affiliations:** 1 Department of Biology, Institute of Molecular Genetics and Cell Biology, Ulm University, D-89081 Ulm, Germany

**Keywords:** Septins, Protein purification, SNAP tag, Protein interactions

## Abstract

**Background:**

The detailed understanding of the functions and mechanisms of the actin and microtubuli cytoskeleton depended, besides innovative methods in live cell imaging, on the purification and labeling of its constituents. This allowed researchers to quantitatively measure filament stability, the rates of filament turnover as well as the determination of the influence of cofactors on filament formation and structure. Septins form the least understood class of cytoskeletal structures in nearly all eukaryotic cells so far examined. In yeast, they comprise a family of proteins (Cdc3, Cdc10, Cdc11, Cdc12, Shs1) that form a co-polymeric, ring-like structure beneath the membrane. This ring serves as a template for the formation of a new bud neck and as a landing pat for proteins involved in polar growth and cytokinesis. Further progress in investigating the mechanisms of septin-structure formation and regulation is hampered by the lack of protocols to modify homogenous samples of purified septins with useful probes for *in vitro* biochemical studies.

**Results:**

We present a protocol for the purification and labeling of yeast septin rods. The four individual septin subunits were co-expressed in *E.coli*. One subunit of the septin polymer was expressed as SNAP tag fusion protein allowing for rapid and stoichiometric labeling with derivatized Benzylguanine (BG). To demonstrate the applicability of our approach, we introduced two different SNAP tag substrates: septin rods labeled with fluorescent BG compounds enabled us to monitor the formation of filaments by fluorescence microscopy whereas BG-biotin was used to couple septin rods to a sensor chip for quantitative surface plasmon resonance binding experiments. In a first application, we determined the affinity and the binding kinetics of the yeast protein Bni5 to the individually coupled septin rods. In a further application we could demonstrate that a once formed septin rod hardly exchange its subunits.

**Conclusions:**

The herein introduced protocol of purifying SNAP tag modified septins from *E.coli* allowed us to derivatize the obtained septin rods with probes for the further *in vitro* characterization of this class of cytoskeletal elements. The availability of a very diverse set of SNAP tag substrates should open the way to investigate different aspects of septin biochemistry in mechanistic detail.

## Background

The classical Hartwell screen revealed, besides many other genes involved in cell cycle regulation of yeast, mutations in the four genes *CDC3*, *CDC10*, *CDC11* and *CDC12.* These were later classified as members of the septins, a new family of proteins in the budding yeast *Saccharomyces cerevisiae*[1]. Another mitotic septin, Shs1, was identified about a decade later [2]. The four essential septins Cdc3, Cdc10, Cdc11 and Cdc12 were subsequently shown to be localized below the plasma membrane at the bud neck, where they initially form a ring at the cortical site of the future bud. This ring expands into an hour-glass shaped collar that splits at the onset of cytokinesis in two rings with the contractile actin ring positioned between them [3]. The septin collar is supposed to define the diffusion barrier between the mother cell and the growing bud [4]. Septins also act as a scaffold for various other proteins involved in cell wall deposition, cell cycle control and cytokinesis [5,6].

All septins are GTPases that share structural similarities with the small GTPases of the Ras family. The central GTPase domain containing conserved nucleotide binding regions as well as switch I and switch II stretches is extended at its N- and C-terminus by further septin-specific structural elements [7,8]. *S. cerevisiae* septins have been expressed and purified from *E.coli*. The purified four essential septins assemble into a linear heterooctameric rod that displays the following order: Cdc11-Cdc12-Cdc3-Cdc10-Cdc10-Cdc3-Cdc12-Cdc11. These septin rods can be induced to form long septin filaments *in vitro* by lowering the salt concentration of a buffer that otherwise keeps the rods stable in solution [9].

From the crystal structure of the human heterotrimeric SEPT2-SEPT6-SEPT7 complex [10], two different contact sites between alternate septin subunits had been deduced: A G-interface build by two G-domains and a NC-interface involving the N- and C-terminal residues of two neighboring septins.

Septins were shown to associate with cellular membranes, actin filaments and microtubules [11,12]. In mammalian cells, septins are required for correct mitotic spindle organization, chromosome segregation, spindle elongation and midbody abscission [13-15]. A potential role in cancerogenesis is under debate [16].

Different approaches are currently used to label a protein with a sensor to monitor certain aspects of its behavior *in vitr*o or *in vivo*. Among those, the SNAP tag represents a very flexible tool to covalently attach different “functionalities” such as fluorescence, affinity or reactive groups to any protein of interest [17,18]. The SNAP-tag technology is based on an engineered version of the DNA repair protein O^6^-alkylguanine-DNA-alkyltransferase [19] which transfers alkyl adducts from the O^6^-position of guanine covalently to its reactive cysteine independent of the nature of the alkyl group. The method has already been widely used for various *in vivo* and *in vitro* applications [20-22]. Particularly, labeling of SNAP tag fused septins with fluorescent Benzylguanine derivatives in living yeast cells already demonstrated that the tag and its label does not interfere with the functions of the septin subunit and its incorporation into rods and filaments [20].

In order to facilitate biochemical studies of the septins *in vitro,* we genetically fused a SNAP tag to one subunit of the yeast septin rod. The *E.coli* expressed SNAP tagged septin rods allowed us to (i) label them with fluorescent compounds to visualize filament formation by microscopy *in vitro* and to (ii) immobilize septin rods via an attached biotin moiety to a surface plasmon resonance (SPR) sensor chip to estimate their stability and to detect and quantify physical interactions between septin rods and their binding partners independently of posttranslational modifications*.*

## Results and discussion

### Purification of SNAP tag modified septin rods from *E.coli*

**Figure 1:**
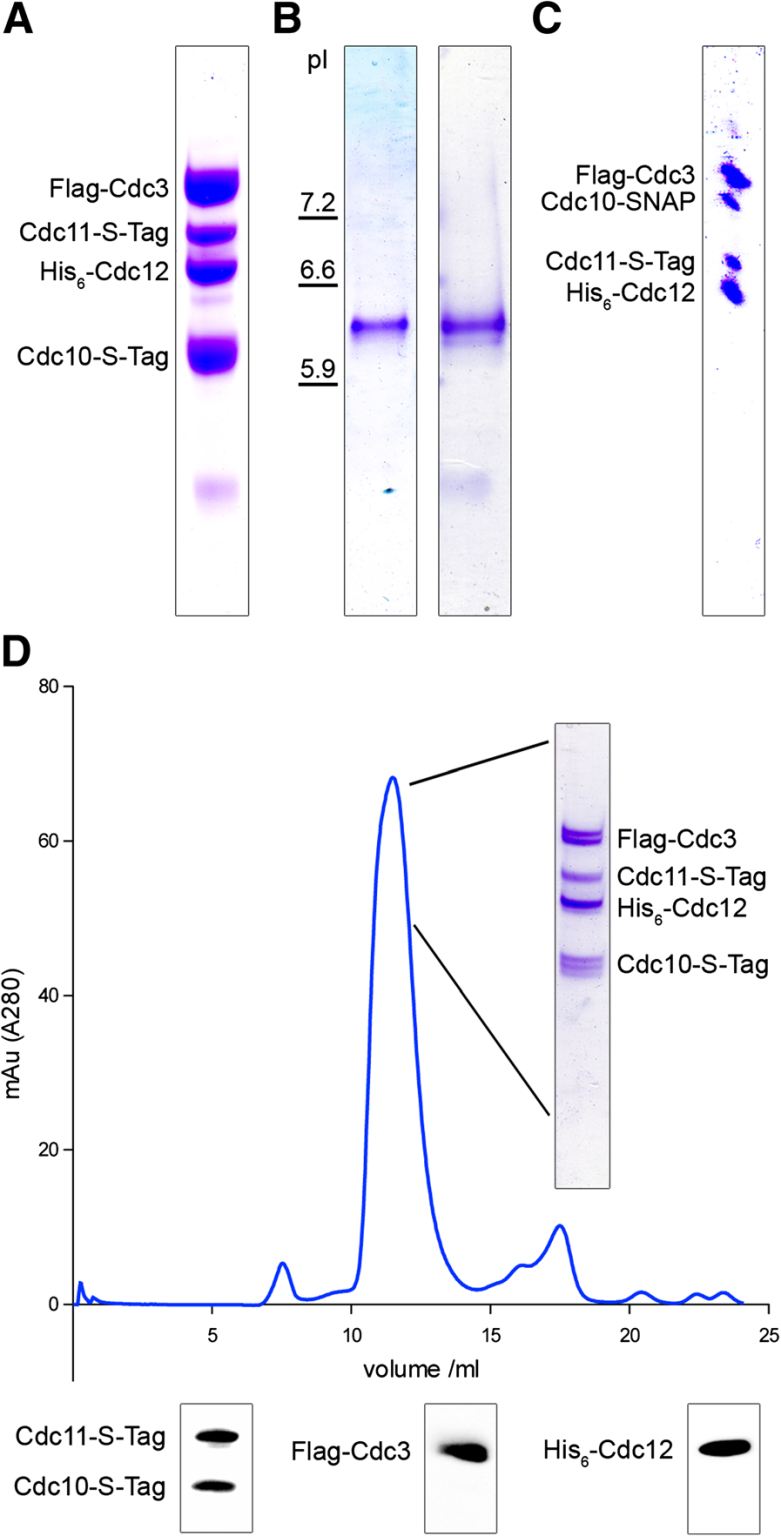
**Purification of recombinant septin rods from *E. coli*. A**. Coomassie stained SDS-PAGE of the purified septin rod. **B**. Isoelectric focussing of purified septin rods (left panel) or SNAP tagged septin rods (right panel). **C**. PAGE separation of the excised band from the IEF for SNAP tagged septin rods. All four septins are present in the complex. **D**. Analytical size exclusion chromatography of purified septin rods. The rods elute in one prominent peak containing all four constituents. The single septins can be detected by Western blot using antibodies against Cdc3 and the attached 6his tag and S tag, respectively.

Before establishing a protocol for the SNAP tagged septin complexes, we first aimed at optimizing the published protocol for the purification of septin rods [9]. We co-expressed the four different septin subunits each fused to a short peptide tag to aid further purification and/or downstream assays: Cdc12 carried the 6-his tag for purification, Cdc10 and Cdc11 carried a S tag and Cdc3 was fused to a N-terminal FLAG tag. Expression was carried out at low temperature over night. As we noticed that recombinant septin rods tended to be unstable in the crude *E.coli* extract, we shortened the purification procedure to less than 6 hours. Specifically, applying a gradient step elution procedure in the IMAC chromatography removed already major contaminants and allowed us to circumvent the time-consuming gel filtration step. The modified protocol yielded around 2.0 mg of intact septin rods per liter expression culture with a purity of >85% (judged by SDS-PAGE; Figure 1A).

We subsequently applied our established purification protocol to SNAP tag modified septin rods. Here, the SNAP tag replaced the S tag of the Cdc10 subunit.

The SNAP tag labeled rods behaved like their S tag labelled counterparts during expression and purification from *E.coli* extracts. To investigate whether the SNAP tag altered the stability and composition of the purified septin rods, we compared the isoelectric points and the aggregation states of the two different samples of purified septin rods (Figure 1B). Both SNAP tagged and untagged rods could be detected as a single band in an IEF gel after Coomassie staining. The estimated pI of 6.0 is in good agreement with the *in silico* predicted pI of 5.8. As the calculated pIs of the isolated septin subunits vary between 4.9 (Cdc11) and 7.8 (Cdc12), a loss of a subunit should have resulted in a readily detectable change in mobility during the IEF analysis. The single band corresponding to the septins was excised from the IEF gel and subjected to denaturing SDS-PAGE. Four bands, corresponding to the four septins, could be detected after Coomassie staining in both septin-rod samples (shown in Figure 1C for SNAP tagged rods). We further investigated the integrity of the purified samples by analytical size exclusion chromatography. Both SNAP tagged and untagged rods eluted in a single peak from the column. The near stoichiometric presence of all four septins in the peak fraction was confirmed by SDS-PAGE and Western blot analysis (Figure 1D).

### Formation of septin filaments *in vitro*

**Figure 2:**
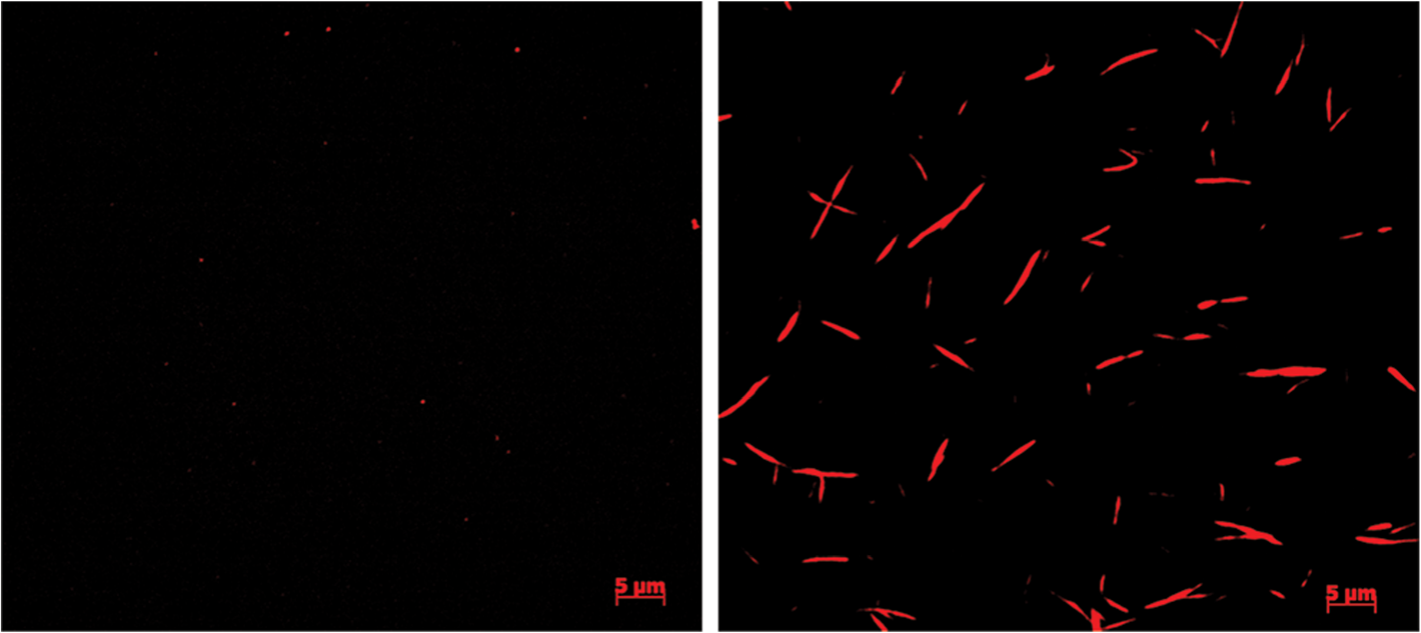
**Fluorescence microscopy of SNAP tagged, TMR labeled septin filaments.** Left panel: No filaments are formed under high salt conditions. Right panel: Filaments in low salt conditions.

**Figure 3:**
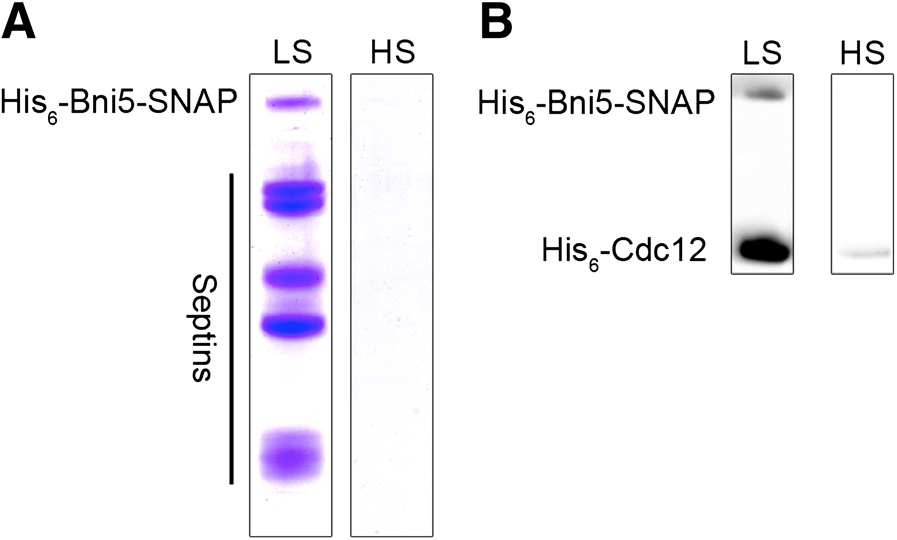
**Bni5 interacts with septins. A**. Spin down assay: SDS-PAGE analysis of supernatant and pellet fraction after centrifugation of a sample containing Bni5-SNAP together with septin filaments (low salt (LS); left panel) or together with septin rods (high salt (HS); right panel). Bni5-SNAP co-sedimentation with the septins indicates interaction. **B**. Validation of the spin down assay with Western blot after SDS-PAGE using an anti-his antibody. 6his tagged Bni5-SNAP is exclusively detected in the pellet with the septins (6his-Cdc12 as control).

Septin filament formation can be induced *in vitro* by lowering the salt of the buffer below 100 mM [9]. A solution containing 2 μM SNAP tagged septin rods in high-salt buffer was labeled with an excess of BG-TMR and subsequently dialyzed into low salt buffer and incubated at 4°C over night. The labeled filaments were then pelleted and captured on poly-L-Lysine coated glass slides for observation under a fluorescence microscope. We observed filaments with lengths ranging from 1.4 to 14.5 μm (Figure 2). Filament formation was strictly suppressed by high salt. To evaluate whether the fusion to the SNAP tag interferes with essential features of the formed filaments, we induced filament formation by dialysis in low salt buffer and added 2 μM purified Bni5-SNAP fusion to the mixture. Bni5 is an already described septin-interacting protein that colocalizes with septin higher-order structures in yeast [23]. The reported interaction data however fell short of demonstrating that Bni5 physically binds to septin filaments. After 60 min of septin/Bni5-SNAP co-incubation, the samples were pelleted and analyzed by SDS-PAGE and Western blot. The appearance of Bni5-SNAP in the pellet fraction was strictly depending on the presence of the septins and only observed under filament-forming conditions thus proving that Bni5 also binds to intact septin filaments (Figure 3A).

### SPR with SNAP tag modified septin rods

The dynamic properties of protein-protein interactions influence the behavior of protein complexes or larger protein interaction networks, and are instrumental in understanding the action of drugs on a certain interaction [24,25]. However, most quantitative data on protein interactions are derived under equilibrium conditions (e.g. FRET or calorimetry). In contrast, chip based biosensors in combination with SPR allow to directly measure the on- and off-rates of protein-protein interactions *in vitro*[26].

The gathered evidence of the native-like properties of the SNAP tagged septin rods encouraged us to couple an affinity handle to the SNAP tag for immobilization of the entire rod to a SPR chip for performing quantitative binding experiments. We labeled SNAP tagged septin rods with BG-biotin in solution and used an anti-biotin antibody as capturing molecule on the surface of the chip. This procedure allowed us to mildly regenerate the chip surface by removing the biotinylated SNAP tag fusion protein and thus enabled us to use one and the same chip for different SNAP tagged septin rod preparations. This is an advantage over published methods that employ chips covalently coupled with BG-NH_2_ substrates. Here SNAP tagged fusions are directly captured from solution, thus avoiding previous labeling [27]. However, regeneration of the surface (i.e. removing the analyte when performing kinetic experiments with increasing analyte concentrations) has to be determined individually for each ligand.

The septin rod was coupled through its central Cdc10 subunit to the surface leaving the more peripheral subunits free to dissociate from the rod. This assay configuration was used to determine the stability of the entire rod. We captured septin rods via the SNAP tag and followed the potential dissociation of its subunits by measuring the decline in response units. The constant response units over hours of washing might either indicate a high kinetic stability of the rod or the dissociation of the peripheral subunits already during the capture procedure (data not shown). The N-terminally fused S tag to the Cdc11 subunit of the rod allowed us to control the integrity of the captured rod preparation by injecting the antibody against the S tag in saturating concentrations. During the washing of the chip surface for two hours, the baseline dropped approximately to the capture level very probably indicating the dissociation of the antibody from the S tag. Dissociation of intact rods from the sensor chip surface is unlikely, as the surface is coated to saturation with the anti-biotin antibody.

**Figure 4:**
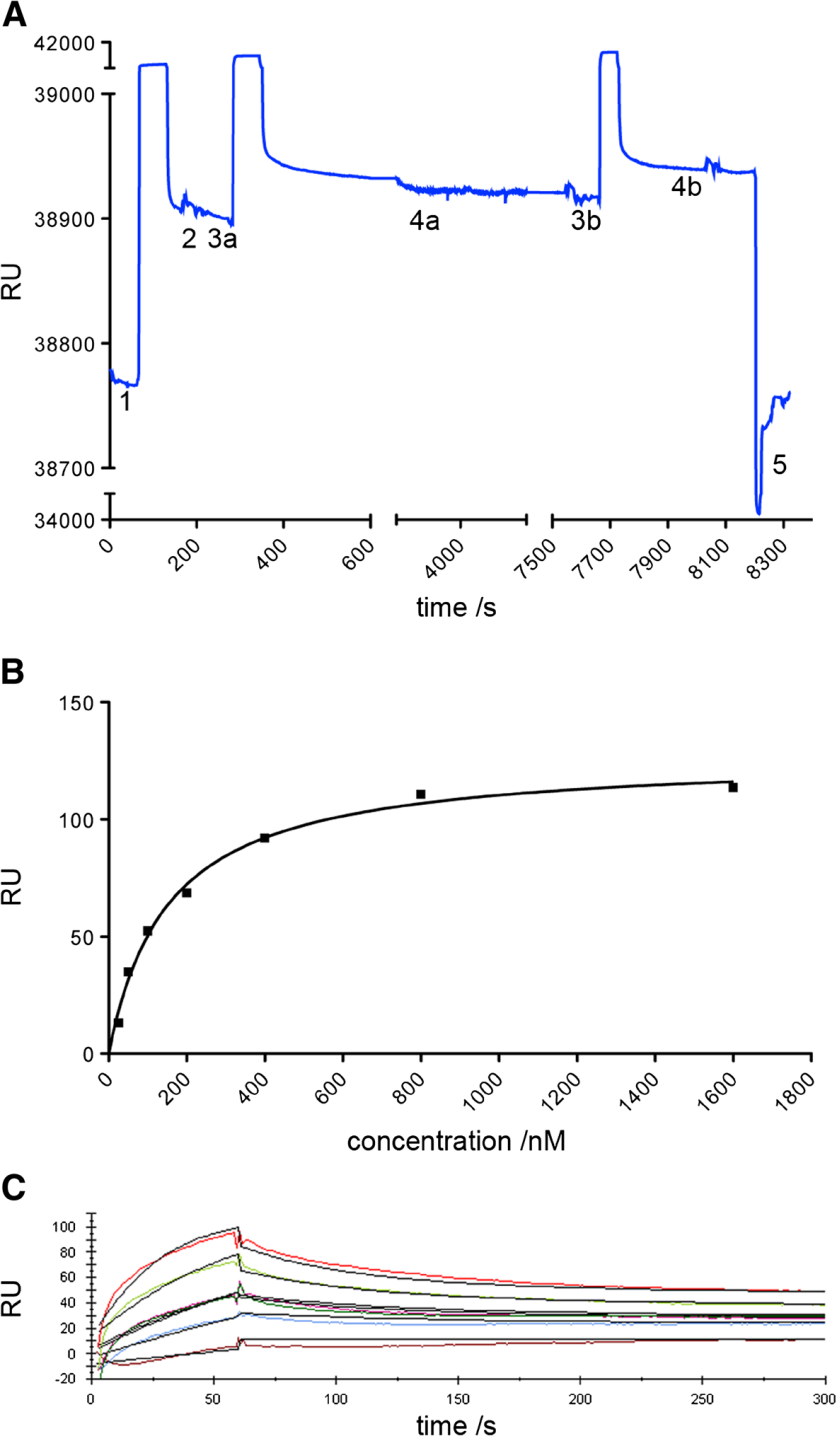
**Analysis of septin rod stability and the interaction of Bni5 with the rod via SPR. A.** Septin rods are stable when immobilized to SPR sensor chips. (1) Capture of biotinylated rods to the anti-biotin antibody coated SPR chip, (2) capture baseline, (3a,b) injection of anti S tag antibody, (4a,b) dissociation, (5) regeneration of the chip surface. **B.** Determination of the K_D_ (3.13x10^-7^ M) of the interaction of Bni5 with immobilized septin rods from the fit of the analyte response plotted against the respective analyte concentration (representative plot from a series of experiments). **C**. Determination of the kinetics parameters from the same dataset as shown in B. The analyte response sensograms were fitted with a two state reaction model to obtain K_D_ (2,53x10^-7^ M), k_on_ (5,1x10^4^ M^-1^s^-1^) and k_off_ (0,037 s^-1^) (representative plot from a series of experiments).

Subsequently, another pulse of anti S tag antibody was injected. The resulting response unit was almost as high as the one obtained after the first injection, indicating the same amount of binding of the antibody to the peripheral Cdc11 subunit and thus the complete integrity of the rod during the whole experiment (Figure 4A).

Using the SPR sensor chip-immobilized septin rods, we next analyzed the interaction between Bni5 and the septins. By plotting the analyte response against the respective analyte concentration, we calculated a dissociation constant of 3.13x10^-7^ M (±1.93x10^-7^, N = 4) for the interaction between Bni5 and septin rods (Figure 4B). A very similar dissociation constant of 2,53x10^-7^ M (±1.37x10^-7^, N = 3) was obtained when we fitted the analyte response sensograms of the experiments with a suitable model (Figure 4C). Here, a two state reaction model was used to compensate for background binding of Bni5 to the antibody-coated chip surface. The on- and off-rates of the interaction were also calculated from a two-state reaction fit to the respective analyte response sensograms (k_off_ = 0,037 s^-1^ ± 0,019, N = 4; k_on_ = 5,1x10^4^ M^-1^s^-1^ ± 0,45x10^4^, N = 3).

To our knowledge these experiments provide the first quantitative description of an interaction between functional septin rods and one of its ligands. Our measurements provide no indication that either phosphorylation of Bni5 [28] or the posttranslational modifications of the septins are a prerequisite for a successful interaction: Bni5 and septins both purified from *E.coli* should lack all posttranslational modifications that are reported to occur in yeast [29,30]. Whether phosphorylation of Bni5 affects the affinity or the kinetics if its interaction with the septins remains to be explored.

## Conclusions

We provide methods for purifying SNAP tag labeled septin rods in high yield and purity. Filament formation and binding assays confirmed that the labeled septin rods display native-like properties. The advantage of the approach was documented by labeling the SNAP tagged septin rods with two different BG-derivatives. Coupling a fluorescent dye allowed to monitor septin filament formation whereas coupling of Biotin allowed immobilizing septin rods on SPR sensor chips. The septin immobilized SPR sensor chips were used to determine for the first time the stability of septin rods and to exemplarily characterize the kinetic and equilibrium constants of an interaction between septin rods and its ligand Bni5.

In contrast to approaches using an individual tag for each application the SNAP tag of one septin preparation can be used for various analytical downstream applications.

The herein introduced methods thus significantly broaden the spectrum of analytical tools to investigate this important but still poorly characterized new class of cytoskeletal structures.

## Methods

### Cloning, expression and purification of septin rods

We aimed at purifying the essential septins Cdc12, Cdc11, Cdc3 and Cdc10 simultaneously from the same cell by cloning each two ORFs (Cdc12/Cdc11 and Cdc3/Cdc10) in one bicistronic expression vector. The four essential septins CDC3, CDC10, CDC11 and CDC12 were amplified by PCR from genomic DNA and cloned in two bicistronic vectors with compatible replicons (pACYC-Duet1 and pET-Duet1, Novagen). A list of all PCR primers used in this work is provided in the Additional file 1.

FLAG-CDC3 (with DYKD coding for the FLAG Tag) and CDC10 fused to the S tag or to an engineered version of the SNAP tag [19] were cloned in pACYC-Duet1 leading to pACYC-Duet1-FLAG-CDC3-CDC10-S or pACYC-Duet1-FLAG-CDC3-CDC10-SNAP respectively. The 6his tag of the first multiple cloning site was removed. CDC12 in frame with the 6his tag and CDC11 fused to the S tag were cloned in pET-Duet1 leading to pET-Duet1-6his-Cdc12-CDC11-S. The plasmids pACYC-Duet1-FLAG-CDC3-CDC10-S or pACYC-Duet1-FLAG-CDC3-CDC10-SNAP and pET-Duet1-6his-CDC12-CDC11-S were co-transformed in the *E. coli* strain BL21(DE3). The transformed cells were grown in Super Broth (SB)-Medium (containing Ampicillin and Chloramphenicol) to an OD_600nm_ of 1.0, induced with 1.0 mM isopropyl-β-D-thiogalactoside for 21 h at 18°C, harvested by centrifugation, washed once with PBS and stored at −80°C. Frozen cells were resuspended in extraction buffer (300 mM NaCl, 2 mM MgCl_2_, 15 mM imidazole, 5 mM β-mercatoethanol, 40 μM GDP, 12% (v/v) glycerol, 0.1% (v/v) Tween20, Complete Protease Inhibitors Cocktail (Roche), 50 mM Tris–HCl pH 8.0) and cell lysis was performed by addition of 1.0 mg/ml lysozyme (30 min on ice) and sonication (Branson Sonifier A250). The resulting lysate was clarified by centrifugation at 40,000 g for 10 min at 4°C and the supernatant was loaded on an ÄktaPurifier Chromatography System (GE Healthcare). The first step of purification was an immobilized metal affinity chromatography (IMAC) by capturing the septin rod via the 6his tag at the N-terminus of Cdc12 on a HisTrap-HP column with 5 ml bed volume (GE Healthcare) equilibrated with IMAC buffer A (300 mM NaCl, 2 mM MgCl_2_, 15 mM Imidazole, 5 mM Dithiothreitol (DTT), 12% (v/v) glycerol, 50 mM Tris–HCl pH 8.0). The proteins were eluted from the column by applying consecutive Imidazole segment gradients with 3%, 25% and 100% IMAC buffer B (500 mM NaCl, 2 mM MgCl_2_, 500 mM Imidazole, 5 mM DTT, 12% (v/v) glycerol, 50 mM Tris–HCl pH 8.0). The septin rods eluted at 25% IMAC B. Septin rod-containing fractions were pooled and buffered in BisTris pH 7.5 (prepared by the Buffer Prep device of the chromatography system) using PD10 desalting columns (GE Healthcare).

The second purification step was performed via a Resource Q anion exchange column (GE Healthcare) in Buffer Prep mode (BisTris pH 7.0) and elution with a linear NaCl gradient. The septin rod eluted at 600 mM NaCl.

The septin-containing fractions were pooled, buffered in Tris-buffer (50 mM Tris–HCl pH 8.0, 300 mM NaCl) containing 10% (v/v) glycerol using PD10 desalting columns and concentrated using Amicon Ultra Centrifugal Units (Millipore). Protein concentration was determined at 280 nm in a NanoDrop ND-1000 spectral photometer (Peqlab) with calculated extinction coefficients (Septin rod: 251.2 mM^-1^cm^-1^, Septin rod-SNAP: 293.5 mM^-1^cm^-1^) and molecular weights (Septin rod: 397.7 kDa, Septin rod-SNAP: 432.5 kDa) or with Bradford [31]. For predictions of the molecular weight and extinctions coefficients the bioinformatic tools provided on the EXPASY server (http://www.expasy.org) were used. Purified complexes were aliquoted and stored at −20°C.

The activity of the SNAP tag was confirmed by labeling the Cdc10-SNAP with BG-TMR (New England Biolabs). The SNAP tagged septin rod was incubated with an excess of substrate for 30 min at RT, separated by SDS-PAGE and TMR fluorescence in the gel was finally visualized through the Cy3-Filter of a Stella Documentation System (Raytest) (data not shown).

Isoelectric focusing (IEF) was performed using precast pH 3–10 IEF gels (Invitrogen) according to the manufacturer’s instructions.

For analytical size exclusion chromatography, purified septin rods were loaded on a Superose 6 10/300 GL column (GE Healthcare) pre-equilibrated with 300 mM NaCl, 50 mM Tris–HCl pH 8.0.

### Expression and purification of Bni5 and Bni5-SNAP

BNI5 from *S.cerevisiae* was PCR amplified from genomic DNA, cloned in a pET15b derived expression plasmid [32] with or without previously inserted SNAP tag and expressed as 6his tagged protein in *E.coli* strain BL21(DE3) in SB medium at 18°C. Purification was done by IMAC, followed by size exclusion chromatography against HBSEP buffer (10 mM HEPES, 150 mM NaCl, 3 mM EDTA, 0.05% Tween 20, pH 7.4) using a Superdex 200 16/60 column (GE Healthcare).

### Analysis of filament formation

Analysis of septin filament formation was performed using an established protocol [33] with some modifications: The purified septin rods were buffered in high salt buffer (300 mM NaCl, 50–200 mM Tris–HCl pH 8.0) using NAP5 columns (GE Healthcare), diluted to 2 μM and clarified by centrifugation at 100,000 g for 1 h at 4°C. The supernatant was subsequently dialyzed (MF-Membrane Filters 0.025 μm; Millipore) into low salt buffer (50 mM NaCl, 50–200 mM Tris–HCl pH 8.0) for 1 h on ice and incubated for 16 h at 4°C.

Septin filaments were subsequently pelleted at 100,000 g for 1 h at 4°C. Filament formation was analyzed by applying the supernatant and the pellet on a SDS-PAGE followed by coomassie staining. BG-TMR labeling of SNAP tagged septin rods was performed in high salt conditions prior to the first centrifugation step by adjusting the septin mixture to 8 μM BG-TMR. Filaments were transferred to poly-L-lysine-coated slides (Science Services), fixed with 3% (w/v) paraformaldehyde for 15 min at RT, blocked with 10% (w/v) bovine serum albumin (30 min at RT), washed with PBS and embedded with VectaShield Hard Set mounting medium (Vector Laboratories). TMR labeled septin filaments were subsequently visualized by fluorescence microscopy using an Axio-Observer Z1 SD confocal microscope (Zeiss). The length of the filaments was determined using the evaluation tool implemented in the aquisition software AxioVision (version 4.8.1, Zeiss).

For the qualitative monitoring of septin filament interaction, 2 μM Bni5-SNAP was added to the reaction after filament formation. The mixture was incubated for 1 h prior to centrifugation (“spin down assay”).

### Analysis of septin-interacting proteins by SPR

Kinetics of septin-interacting proteins were measured by surface plasmon resonance using a Biacore X100 system (GE Healthcare).

For the capture of biotinylated septin rods, the surface of a CM5 Chip (GE Healthcare) was previously coated with an anti-Biotin-antibody (US Biologicals) as capture molecule using NHS ester chemistry in 10 mM Sodium actetate pH 5.0 with HBSEP as running buffer.

Purified SNAP tagged septin rods (ligand protein) were covalently labeled with BG-Biotin (New England Biolabs) by SNAP tag chemistry in MSP buffer (10 mM MES, 150 mM NaCl, 0.05% Tween 20, pH 7.4) adjusted to 300 mM NaCl. Excess substrate was removed using a NAP5 desalting column.

MSP was used as running buffer for all subsequent experiments. Capture levels for the ligand were in the range of 100 RU.

For determination of the rod stability, an anti-S tag antibody (Novagen; 20 μg/ml in MSP buffer) was injected via a 60 s pulse after ligand capture and the chip surface system was washed for 2 h with running buffer before another 60 s pulse of antibody was injected.

The anti-S tag antibody did not show significant binding to the sensor chip surface coated with anti-biotin antibody (Additional file 2: Figure B).

For the determination of kinetic parameters, purified Bni5 (analyte protein) was prepared in suitable concentrations (0–1600 nM) in MSP buffer. The contact time with the ligand was set to 120 or 180 s followed by a 600 s dissociation period for each cycle. SPR detection was performed simultaneously on a reference cell (with capture molecule) and a detection cell with capture molecule and ligand. Background correction (i.e. substraction of the reference cell signal) is performed by default.

Regeneration of the sensor chip between each analyte cycle was performed with a 15 s injection pulse of 12 mM NaOH. Kinetic constants were calculated with the Biacore Evaluation Software (Version 1.1; GE Healthcare).

## Abbreviations

BG: Benzylguanine; IMAC: Immobilized Metal Affinity Chromatography; TMR: Tetramethylrhodamine; MW: Molecular weight (kDa); SPR: Surface plasmon resonance.

## Competing interests

The authors declare that they have no competing interests.

## Authors’ contributions

CR performed the experiments and prepared the figures. TG performed SPR measurements and wrote with contributions of NJ and CR the manuscript. NJ oversaw the project and helped to analyze the data. TG, NJ and CR conceived of the study. All authors read and approved the final manuscript.

## Supplementary Material

Additional file 1 List of PCR primers used in this study. Click here for file

Additional file 2 **SPR control experiments.** A. SNAP tagged, unlabeled septin rods are injected on a sensor chip without previous ligand capture. No significant background binding to the capturing molecule can be detected. B. Anti-S tag antibody is injected on a sensor chip without previous ligand capture. No significant background binding to the capturing molecule can be detected. Click here for file
