# Supplementary figures and images for: An efficient protocol for the purification and labeling of entire yeast septin rods from E.coli for quantitative in vitro experimentation
Source: BMC Biotechnol. 2013 Jul 26;13:60. doi: 10.1186/1472-6750-13-60 (PMC3765318; doi:10.1186/1472-6750-13-60)

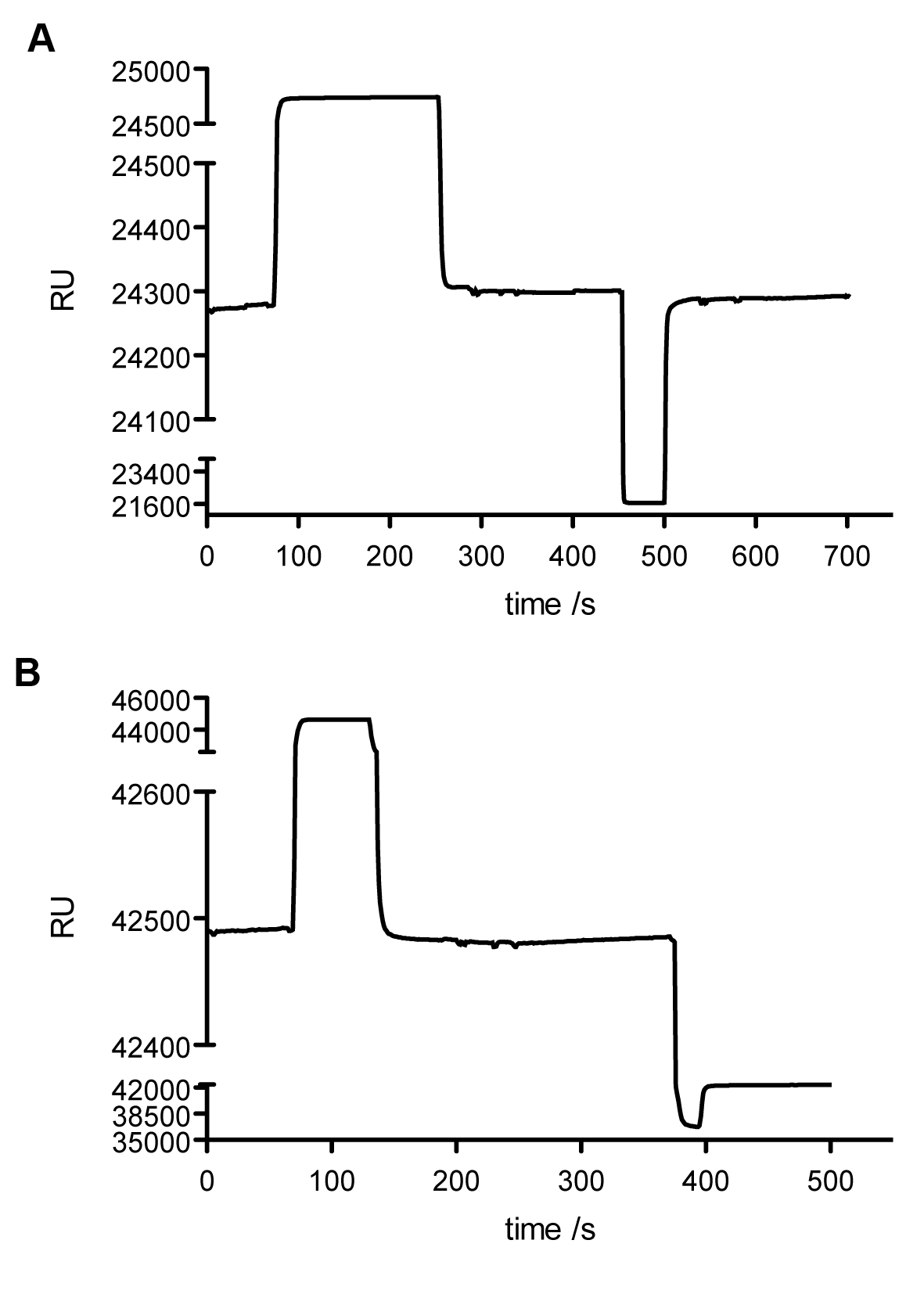

Supplement: Additional file 2 — SPR control experiments. A. SNAP tagged, unlabeled septin rods are injected on a sensor chip without previous ligand capture. No significant background binding to the capturing molecule can be detected. B. Anti-S tag antibody is injected on a sensor chip without previous ligand capture. No significant background binding to the capturing molecule can be detected. [file 1472-6750-13-60-S2.tiff]
